# Miconazole alleviates peripheral nerve crush injury by mediating a macrophage phenotype change through the NF‐κB pathway

**DOI:** 10.1002/brb3.1400

**Published:** 2019-09-04

**Authors:** Liangliang Zhang, Xiuju Chen, Zengyun Liu, Qingluan Han, Liguo Tang, Zhen Tian, Zhiyong Ren, Cunmin Rong, Hui Xu

**Affiliations:** ^1^ Department of Hand Surgery Affiliated Hospital of Jining Medical University Jining Shandong China; ^2^ Jining Medical University Jining Shandong China; ^3^ Department of Neurology Tianjin Nankai Hospital Tianjin China; ^4^ Department of Orthopaedic Sunshine Union Hospital Weifang Shandong China

**Keywords:** macrophage, miconazole, NF‐κB, peripheral nerve injury, polarization

## Abstract

**Background:**

Peripheral nerve injury (PNI) causes motor and sensory defects, has strong impact on life quality and still has no effective therapy. Miconazole is one of the most widely used antifungal drugs; the aims of the study were to investigate the effects of miconazole during sciatic nerve regeneration in a mouse model of sciatic nerve crush injury.

**Methods:**

We established peripheral nerve crush model and investigated the effects of miconazole by multiple aspects. We further studied the potential mechanism of action of miconazole by Western blotting, fluorescence immunohistochemistry, and PCR analysis.

**Results:**

Miconazole improves the symptoms of crushed nerve by improving inflammatory cell infiltration and demyelinating myelin of sciatic nerve. Affected by miconazole, the proportion of inflammatory M1 macrophages in the distal part of the sciatic nerve was reduced, and the proportion of anti‐inflammatory M2 macrophages was increased. Finally, the neuroprotective properties of miconazole may be regulated by the nuclear factor (NF)‐κB pathway.

**Conclusions:**

Our data suggest that miconazole can effectively alleviate PNI, and the mechanism involves mediating a phenotype change of M1/ M2 macrophages. Thus, miconazole may represent a potential therapeutic intervention for nerve crush injury.

## Graphical abstract

Miconazole protects against sciatic nerve crush injury. Miconazole promotes M2 macrophage polarization in vivo and in vitro. Miconazole‐related protection is mediated by the NF‐κB pathway.

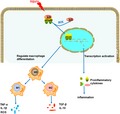

## 1 INTRODUCTION

Peripheral nerve injury (PNI) is mainly caused by bone dysplasia, trauma, birth trauma, chemical poisons, etc., including the interruption of nerve continuity and nerve cell damage. Peripheral nerve injury (PNI) results in pathological pain and muscular dystrophy, spasticity, and motor disability. The mechanisms that cause neuropathic pain and fail to reconstruct neuromuscular synapses are not fully understood. The demyelinating diseases and trauma of peripheral nervous system (PNS) lead to action potential defects and even damage motor function (Altmann et al., 2016; Bombeiro et al., 2016). The incidence of peripheral nerve injury (PNI) is higher than that of the central nervous system, but is not as well studied (Yuan & Feng, 2016). Indeed, the repair and regeneration of peripherally injured nerves is an extremely complex process. PNI around the damaged microenvironment causes a series of local responses, which to a certain extent, affects the degenerated nerve regeneration (Freria et al., 2016). Peripheral nerve injury results in degeneration of damaged axons and their myelin sheaths distal to the site of the injury, a process called Wallerian degeneration (WD; Mietto, Mostacada, & Martinez, 2015). Wallerian degeneration is one of the most important processes involved in PNI. It induces multiple changes, such as glial cell proliferation, axonal degeneration, myelin disintegration, blood–nerve barrier (BNB) compromise, even the infiltration and activation of immune cells. When axons in the PNS are injured, the distal portion progressively degenerates (Wallerian degeneration), a process that is produced by the breakdown of both axons and myelin 1. However, PNS is not completely isolated, and the injured axons trigger a complex multi‐cellular response that involves multiple components (DeFrancesco‐Lisowitz, Lindborg, Niemi, & Zigmond, 2015; Gaudet, Popovich, & Ramer, 2011).

In addition, the inflammation plays a key role in a series of pathological reaction of neurological disease. Complex correlations between Schwann cells, macrophages, and axons during nervous demyelinating and myelin regeneration (Jiang, Li, et al., 2014; McLean & Verge, 2016; Niemi et al., 2013). Inflammatory response is a hallmark of demyelinating pathology. Macrophages are one of the most notable types of immune cells that play a key role in nerve injury and repair during this process (Chen, Piao, & Bonaldo, 2015; McLean & Verge, 2016; Stratton & Shah, 2016). When a peripheral nerve is injured, macrophage quickly changes its functional phenotype in response to the microenvironment and begins to accumulate at the injury site within 2–3 days, peaking at 7 days after injury. The stages of macrophage activation are the “classically activated” proinflammatory M1 macrophages that contribute to Wallerian degeneration, and the “alternatively activated” anti‐inflammation M2 macrophages that contribute to axonal regeneration (Chen, Cescon, et al., 2015; Chen, Piao, et al., 2015; Stratton & Shah, 2016). After being stimulated by interferon (IFN)‐γ or lipopolysaccharide (LPS), macrophages can be transformed into an M1 phenotype secreting tumor necrosis factor (TNF)‐α, inducible nitric oxide synthase (INOS), interleukin (IL)‐1β, interleukin (IL)‐6, and cyclooxygenase (COX)‐2. A typical phenotype of high expression of (IL)‐1β, interleukin (IL)‐6, and cyclooxygenase (COX)‐2. Conversely, macrophages can shift to an M2 phenotype with the stimulation of IL‐4, a condition characterized by high expression of arginase I (Arg‐1), mannose receptor C type 1 (MRC1/CD206), and transforming growth factor (TGF)‐β (Chen & Bonaldo, 2013; Mokarram, Merchant, Mukhatyar, Patel, & Bellamkonda, 2012). The expression of these molecules triggers the recruitment and activation of macrophages from the peripheral circulation and the proliferation and activation of resident macrophages (Fry, Ho, & David, 2007; Perrin, Lacroix, Aviles‐Trigueros, & David, 2005). However, the exact mechanisms governing macrophage polarization in peripheral nerve injury model are still poorly understood.

Miconazole is an antifungal medication and received FDA approval in 1974, it has been used dermatologically or vaginally to treat fungal infections. Miconazole is an important drug clinically used to inhibit the growth of fungi. In previous studies, this drug was only approved for fungal infections. However, some studies have reported that miconazole promotes myelination in toxin‐induced spinal cord lesions (Cully, 2015). It has also been reported that miconazole can promote oligodendrocyte progenitor cell regeneration and enhance myelination in experimental autoimmune encephalomyelitis (EAE). However, the therapeutic potential of miconazole for PNI and the precise mechanism are unknown. Therefore, we pursued our hypothesis that miconazole may prevent overstimulation of immune responses, thereby inhibiting macrophage differentiation and cytokine production to exert anti‐inflammatory and neuroprotective effects in PNI and tried to investigate the probable pathway.

## 2 MATERIALS AND METHODS

### 2.1 Animals and surgical procedure

A total of 300 healthy male C57BL/6 mice (Charles River Laboratory) weighing 20–25 g and aged 10–12 weeks were housed under conventional experimental environment with 12‐hr light–dark cycle in the Animal Care Facility. Prior to this study, these mice were given free access to commercial‐standard mouse diets and water. All experiments were carried out in accordance with the Nankai Hospital Animal Care and Use Committee (NKYY‐DWLL‐2019‐057) Agreement established by the Chinese Animal Protection Committee.

Preoperatively, in order to determine the effective concentration of miconazole in the experimental mice, we performed a dose‐dependent pre‐experiment. In the preliminary trial, 30 mice were randomly divided into five subgroups (*n* = 6), and then each group received an injection of 0, 5, 10, 20, and 40 mg/kg of miconazole. In formal experiment, mice were randomly divided into three groups: a sham‐operated group (*n* = 30), a control group (*n* = 30), and a miconazole treatment group (*n* = 30). Postoperation, each group of mice was randomly divided into five parts according to different treatment methods: Assessment of walking tracks (*n* = 6), the Electrophysiological (*n* = 6, 7 and 28 days postoperation), Histopathological assessment (H&E and LFB) and Fluorescence immunohistochemistry and analysis (*n* = 6), Real‐time quantitative polymerase chain reaction (RT‐PCR) and Western blot analysis (*n* = 6). We applied a sample size calculation (beta error level 20% and alpha error level 5%) and our previous experience, resulting in a sample size of six mice per group. All the experiments were repeated three times.

Prior to surgery, functional healthy state of all animals was assessed by means of walking tracks. All surgeries and electrophysiological measurements were performed under aseptic conditions. For sciatic nerve crush injury, 10‐ to 12‐week‐old C57BL/6 mice were anesthetized with ketamine and xylazine. The sciatic nerve crush operation was performed using methods previously reported (Bombeiro et al., 2016) and the surgeon was blinded to the group identity. Briefly, under anesthesia with ketamine (100 mg/kg) and xylazine (20 mg/kg), a skin incision was made 10 mm below and parallel to the left femur, the left sciatic nerve was exposed and crushed at mid‐thigh level for 30 s using a fine forceps (#5C; Fine Science Tools), as described previously (Mietto et al., 2013; Siqueira Mietto et al., 2015). Then the incision was closed with absorbable sutures. Mice underwent the same surgical procedure without constant pressure from the forceps referred as the sham‐operated group.

Sham‐operated group, exposure of the sciatic nerve (*n* = 30);
Control group, exposure of the sciatic nerve + crush injury (*n* = 30);
Miconazole treatment group, exposure of the sciatic nerve + crush injury (*n* = 30).

To keep the body temperature of mice within the normal range (37–38°C) with a temperature‐controlled heating pad. Food and water were provided ad arbitrium until the mice fully gained consciousness. For analgesia, buprenorphine (0.03 mg/kg) was administered i.p. every 12 hr for the first 24 hr.

### 2.2 Treatment with miconazole

**Figure 1:**
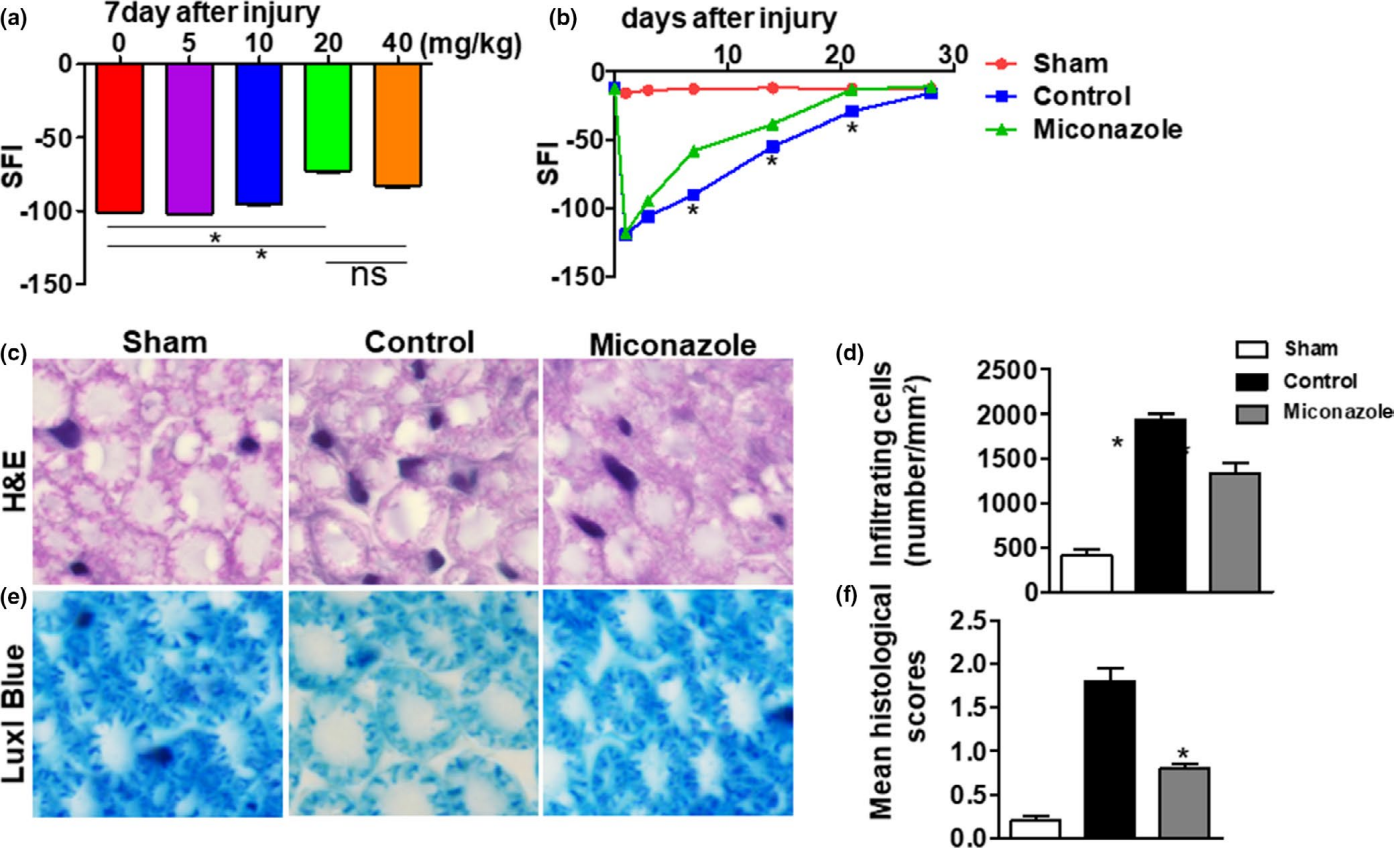
Miconazole treatment ameliorates the SFI, histological changes, and inflammatory cell accumulation in PNI. (a) Miconazole dose‐dependently reduced the SFI at 7 days after nerve crush, and the optimum dose to produce this effect is 20 mg/kg. (b) Analysis of the SFI after nerve crush with or without a 20 mg/kg dose of miconazole. Miconazole‐treated mice exhibited significantly better clinical features from day 7 to day 28 compared with the mice in the control group. (c) On day 7 postinjury, sciatic nerves of each group were harvested for H&E staining (c) and LFB staining (e) to assess changes in infiltration and myelin degeneration. Representative photomicrographs are shown for each group. The mean number of inflammatory cells per square millimeter of tissue section was determined as described in the Section 2, and summaries are shown in d and f. Comparisons between the control group and miconazole‐treated group were performed using Mann–Whitney *U* tests. The results are presented as the means ± *SEM* (*p < .05, *n* = 6 per group)

Miconazole (0765, Sigma‐Aldrich) was suspended in 5% dimethylsulfoxide (DMSO). To determine the appropriate dose for treatment, 30 mice were assigned to five groups (*n* = 6) on the basis of receiving 0, 5, 10, 20, and 40 mg/kg of miconazole randomly. For our experiment, the dose which could alleviate the injury to the most extent was 20 mg/kg (Figure 1a). Therefore, the dose of miconazole used in our next experiment was determined to be 20 mg/kg. Our study included three groups: a sham‐operated group (*n* = 30) (vehicle‐treated), a control group (*n* = 30) [PNI + phosphate‐buffered solution (PBS)], and a miconazole treatment group (*n* = 30) (PNI + miconazole). In the miconazole treatment group, the mice were given the dose of 20 mg/kg miconazole 48 hr after the animal operation, and once daily, continued for 5 days until the sciatic nerve was removed on the 7th day after surgery for the corresponding histology. In the sham‐operated and control groups, the mice were given equivalent volume of PBS. All mice were given an intraperitoneal injection of the same dose.

### 2.3 Assessment of walking tracks following nerve crush injury

All mice underwent walking track analysis 7 days before crush injury and 1, 3, 7, 14, 21, and 28 days after the operation, as previously described (Bain, Mackinnon, & Hunter, 1989; Inserra, Bloch, & Terris, 1998; Yuan et al., 2010), with a minor adjustment. The hind paws painted with blue ink were used to record the paw prints and allowed the mice to walk along a 6 cm × 40 cm on white paper (Islamov et al., 2002). For measurement, clear and steady prints were recorded when the mouse was walking at a smooth pace. Three different parameters were evaluated in our context: toe spread (TS), the distance between first and fifth toes; intermediate toe spread (IT), the distance between the second and fourth toes; print length (PL), the distance between the third toe and the hind pad. Measurements of all parameters were made for both normal and the experimental paw prints. The sciatic functional index (SFI) was calculated as (Bain et al., 1989; Inserra et al., 1998; Islamov et al., 2002) SFI = −38.3 [(EPL‐NPL)/NPL] + 109.5 [(ETS‐NTS)/NTS] + 13.3 [(EIT‐NIT)/NIT] − 8.8, in which ETS is experimental toe spread, NTS is normal toe spread, EPL is experimental paw length, and NPL is normal paw length, EIT is experimental intermediate toe spread, NIT is normal intermediate toe spread. The value of SFI decreased to −100 after PNI. As the SFI value approaches zero, the corresponding functional recovery is better (Bain et al., 1989; Chang, Shah, Lee, & Yu, 2018).

### 2.4 Electrophysiological studies

Electrophysiological studies were performed on day 7 and day 28 after sciatic nerve injury, and electromyography (EMG) of the injured sciatic nerve was recorded using a full digital keyboard compact EMG/NCS/EP recording system (Dantec Co). The conductor was blinded to the experiment as previously described (Quintes et al., 2016). First, mice were anesthetized with ketamine (100 mg/kg, i.p.) and xylazine (20 mg/kg, i.p.). In order to record the signals, monopolar needle electrodes were used to stimulate the left sciatic nerve. Left sciatic nerve was exposed by microinstruments from the hip (referred as proximal) to the ankle (referred as distal) very carefully, two pairs of needle electrodes were inserted at the sciatic notch and ankle, respectively. The motor nerve conduction velocity (MNCV) and compound muscle action potential (CMAP) were recorded. Based on the obtained CMAP curve, the value from the baseline to the maximum peak is calculated to determine the magnitude of the amplitude. The latency difference was calculated according to MNCV. The incision was sutured under an aseptic environment after all the measurements were completed. The body temperature of the mice during electrophysiological measurements was maintained within a normal range (37–38°C) by positioning a heating pad under the mouse.

### 2.5 Histopathological assessment

At day 7 after injury, six mice from each group were anesthetized by ketamine and xylazine and were then perfused with 4°C PBS through the left ventricle. We quickly removed the distal stumps of the left sciatic nerve and fixed it overnight at 4°C in 4% paraformaldehyde, lastly embedded in paraffin as previously described (Han, Xiao, Zhai, & Hao, 2016). Serial transverse sections were cut into 8 μm and stained with hematoxylin–eosin (H&E) (Solarbio Science & Technology) and luxol fast blue (LFB), which is a solution of 0.1% LFB, 0.1% cresyl echt violet, and 0.05% lithium carbonate. Images of H&E‐stained and LB tissues were captured using a Nikon digital microscope (Nikon). For the HE sections, the infiltrated cells were presented as the cell numbers per square millimeter (200 × magnification). To test the degree of myelin degeneration, histological scores were counted in line with the following semiquantitative pathological/histological scale: 0 = normal vascular tissue area; 1 = mild cell infiltration close to the vessel; 2 = cell infiltration plus myelin degeneration in directly adjacent to the blood vessel; 3 = cell infiltration and myelin degeneration through the whole section. The observers were blinded to the experiment and all the images were acquired from three random microscopic field.

### 2.6 Fluorescence immunohistochemistry and analysis

On the 7th day, the sciatic nerves were removed, fixed with 4% paraformaldehyde overnight, and then dehydrated sequentially with 15% and 30% sucrose. When the tissue sank to the bottom, nerves were embedded in Tissue‐tek medium (SAKURA) and snap‐frozen in liquid nitrogen to expose antigenic sites for staining. Transverse sections (8 μm) were prepared on a cryostat (Leica Microsystems LM3050S). Fluorescence immunohistochemistry of the frozen sections was performed using standard protocols provided by the antibody manufacturers. Primary antibodies included the following: goat anti‐CD206 (1:200; R&D Systems), rat anti‐CD16/32 (1:500; BD), and rabbit anti‐Iba‐1 (1:500; Wako). Images were taken using a fluorescence microscope (Olympus PX51) and analyzed with Adobe Photoshop 5.0 software. Data are expressed as the percentage of Iba‐1+ cells also positive for CD16/32 or CD206.

### 2.7 Real‐time quantitative polymerase chain reaction

Total RNA was extracted from the left sciatic nerve using Trizol reagent (Invitrogen) according to the manufacturer's instructions. A Nanodrop2000 was used at 260/280 nm to quantify the concentration of RNA. cDNA was transcribed using a Trans‐Script First‐Strand c‐DNA Synthesis SuperMix Kit (Transgen; Catalog: AT301). PCR was performed on an Opticon 2 Real‐Time PCR Detection System (Bio‐Rad) with SYBR gene PCR Master Mix (Roche). The samples were run in duplicate and normalized to glyceraldehyde phosphate dehydrogenase (GAPDH). Then, the expression levels of mRNA were reported using the 2^−ΔΔCt^ method as fold changes versus the sham group. The primer sequences used to amplify genes of interest are listed in Table 1.

**Table 1:** Primers for real‐time polymerase chain reaction

Gene	Primer
GAPDH	Sense: GCCAAGGCTGTGGGCAAG GT
	Reverse: TCTCCAGGCGGCACGCA GA
M1	
CD32	Sense: AATCCTGCCGTTCCTAC TGATC
	Reverse: GTGTCACCGTGTCTTCCTTGAG
CD16	Sense: TTTGGACACCCAGATGTTTCAG
	Reverse: GTCTTCCTTGAGCACCTGGATC
IL‐1β	Sense: ACGCTTACCATGT GAGCTG
	Reverse: GCCACAGGGATTTTGTCGTT
IL‐6	Sense: CCGGAGAGGAGACTTCACAG
	Reverse: TGGTCTTGGTCCTTAGCCAC
TNF‐α	Sense: TCGGTCCCAACAAGGAGGAG
	Reverse: GGGTTGTCACTCGAGTTTTG
M2	
CD206	Sense: CAAGGAAGGTTGGCATTTGT
	Reverse: CCTTTCAGTCCTTTGCAAGC
Arg‐1	Sense: TCACCTGAGCTTTGATGTCG
	Reverse: CTGAAAGGAGCCCTGTCTTG
IL‐10	Sense: AAATAAGAGCAAGGCAGTGG
	Reverse: GTCCAGCAGACTCAATACACA
TGF‐β	Sense: TGCGCTTGCAGAGATTAAAA
	Reverse: CGTCAAAAGACAGCCACTCA

### 2.8 Western blot analysis

On the 7th day, left sciatic nerve was placed in a ristocetin‐induced platelet aggregation (RIPA) buffer which contained protease and phosphatase inhibitors, and homogenized by sonication. For cytoplasmic and nuclear protein extraction, sciatic nerve tissue or cultured cells were first mixed with Buffer A (KeyGene Biotech) and resuspended using a shaker. After 10 min on ice, Buffer B (KeyGene Biotech) was added and the solution was mixed well. Cytoplasmic proteins were obtained by centrifugation at 12,000 *g* at 4°C for 30 s. Afterward, prechilled Buffer C (KeyGene Biotech) was added to the precipitation and the solution was mixed well. After 30 min on ice, nuclear proteins were harvested by centrifugation at 12,000 *g* at 4°C for 10 min. Protein concentrations were evaluated using a Protein Assay Kit (Beyotime), and 15 μg protein was loaded in each lane. Proteins were separated on a 12% gel at 80 V for 30 min and then 120 V for 60 min. After that, the proteins were transferred onto a nitrocellulose membrane for 1.5 hr at 4°C with a current of 80 V. The membranes were blocked with 5% skim milk for 1 hr at room temperature, incubated with primary antibody at 4°C overnight, washed three times at room temperature for 20 min, incubated with HRP‐conjugated secondary antibody (1:5,000; Transgen) for 2 hr, washed three times at room temperature, 20 min each time. Finally, the images were catched by chemiluminescence and quantified by densitometry (Bio‐Rad). All cultures were placed on the presence of 0.1% Tween‐20 and 5% fat‐free dry milk powder dissolved in tris‐buffered saline tween (TBST) buffer. The following primary antibodies were used: rabbit anti‐IκB, rabbit anti‐p‐IκB (1:500; Abcam), and mouse anti‐GAPDH (1:1,000; Sigma).

### 2.9 Cell culture and treatment

Raw246.7 cells are derived from the murine primary macrophage cell line. Primary macrophages were cultured in complete RPMI 1640 medium (Life Technologies) supplemented with 10% fetal bovine serum (FBS; Gibco), 100 U/ml penicillin, and 100 μg/ml streptomycin (Gibco) and placed it in a humidified cell culture incubator at 37°C. For lipopolysaccharide (LPS) stimulation, RAW246.7 cells in culture were hatched with LPS (100 ng/ml; Sigma) in the presence or absence of miconazole (200 ng/ml; Millipore) for 12 hr. RAW246.7 cells were then polarized into the M1 or M2 phenotype with LPS (100 ng/ml; Sigma) or IL‐4 (20 ng/ml; Peprotech), respectively, by incubation for 12 hr with or without miconazole.

### 2.10 Statistical analysis

GraphPad Prism software (GraphPad Software, Inc.) was used. Data are presented as means ± *SEM*. The Mann–Whitney *U* test was used to compare the clinical scores. The two‐tailed Student's *t* test was applied for comparisons between two groups. *p* < .05 was taken as a significant difference.

## 3 RESULTS

### 3.1 Effects of miconazole on SFI and neuroinflammation in PNI mice

Sciatic functional index is a reliable way to estimate the injury and recovery of the innervation of foot muscles, and we first assessed SFI after sciatic nerve crush injury. In the control group, the SFI decreased to a value of −120 at day 1 after injury and this indicated total loss of motor innervation of the foot and then recovered to baseline after 4 weeks (Figure 1b). In miconazole treatment group, the severity of PNI was reduced compared with control group as measured by SFI, and the most significant difference between the miconazole treatment group and the control group was observed at day 7 after injury (Figure 1b). In the miconazole treatment group, mice exhibited significantly better clinical features from day 7 to day 28 compared with the mice in the control group (*p* < .05 at each time point; Figure 1b). At the time of the clinical course when the maximal difference between the control and miconazole treatment groups was observed (i.e., day 7 postinjury), we observed the histopathological changes of the injured sciatic nerves in mice. H&E staining and LFB staining were used to show inflammatory cell infiltration and myelin degeneration, respectively. Treating mice with miconazole decreased the number of inflammatory cells (Figure 1c,d). The incidence of degeneration was also reduced by miconazole treatment (Figure 1e,f).

### 3.2 Changes of sciatic nerve electrophysiology in SFI mice after miconazole treatment

**Figure 2:**
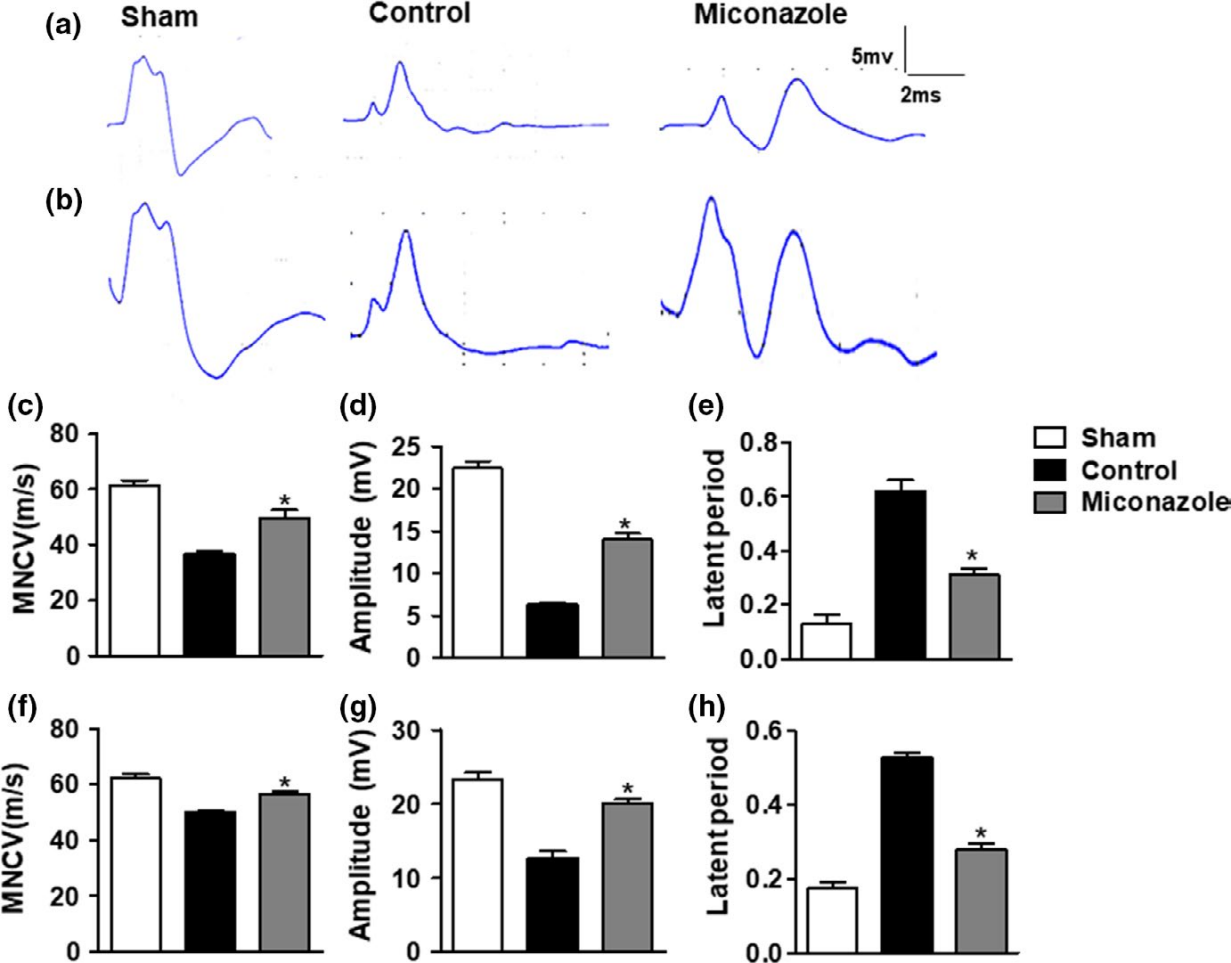
Miconazole protects against sciatic nerve crush injury. (a, b) Representative electrophysiological recordings of motor nerve compound muscle action potentials (CMAPs) evoked from stimulation of ankle regions of the sciatic nerve are shown for sham, control, and miconazole mice, as indicated. Day 7 postinjury (a, c, d, e); Day 28 postinjury (b, f, g, h). (c, f) Compared to the control group, miconazole‐treated nerves were protected from crush‐induced damage of mean motor nerve conduction velocity (MNCV). (d, g) Miconazole treatment improved the amplitude of CMAPs when compared to the control group. (e, h) Compared to the control group, the miconazole treatment group also exhibited better distal motor latencies of CMAPs. Comparisons between the control group and miconazole‐treated group were performed using Mann–Whitney *U* tests. The data are shown as the means ± *SEM* (*p < .05; *n* = 6)

The feature of miconazole to protect sciatic nerve injury was assessed by response electrophysiology in the sciatic nerve at day 7 and 28 postinjury. It is well known that once the sciatic nerve has been injured, the CMAP amplitude and MNCV decrease and CMAP latency is delayed (Ghayour, Abdolmaleki, & Behnam‐Rassouli, 2016). In consistent with the clinical results shown in Figure 1, miconazole treatment alleviated the degree of injury in mice compared to the control group (Figure 2). The mean conduction velocity of the control group was much slower than that of the miconazole‐treated group (day 7:36.67 ± 1.202 vs. 49.67 ± 2.906 m/s, *p* < .05, Figure 2c; day 28:50.00 ± 0.577 vs. 56.67 ± 0.881 m/s, *p* < .05, Figure 2f). The mean CMAP amplitude of the miconazole treatment group was better than that of the control group (day 7:6.23 ± 0.318 vs. 13.97 ± 0.751 mV, *p* < .05, Figure 2d; day 28:12.67 ± 0.877 vs. 20.00 ± 0.581 mV, *p* < .05, Figure 2g). Additionally, miconazole also alleviated CMAP latency, which was shorter in the miconazole treatment group than the control group (day 7:0.62 ± 0.042 vs. 0.31 ± 0.018 ms, *p* < .05, Figure 2e; day 28:0.53 ± 0.014 vs. 0.28 ± 0.015 ms, *p* < .05, Figure 2h).

### 3.3 Miconazole's effects on myelinated nerve fibers after sciatic nerve injury

**Figure 3:**
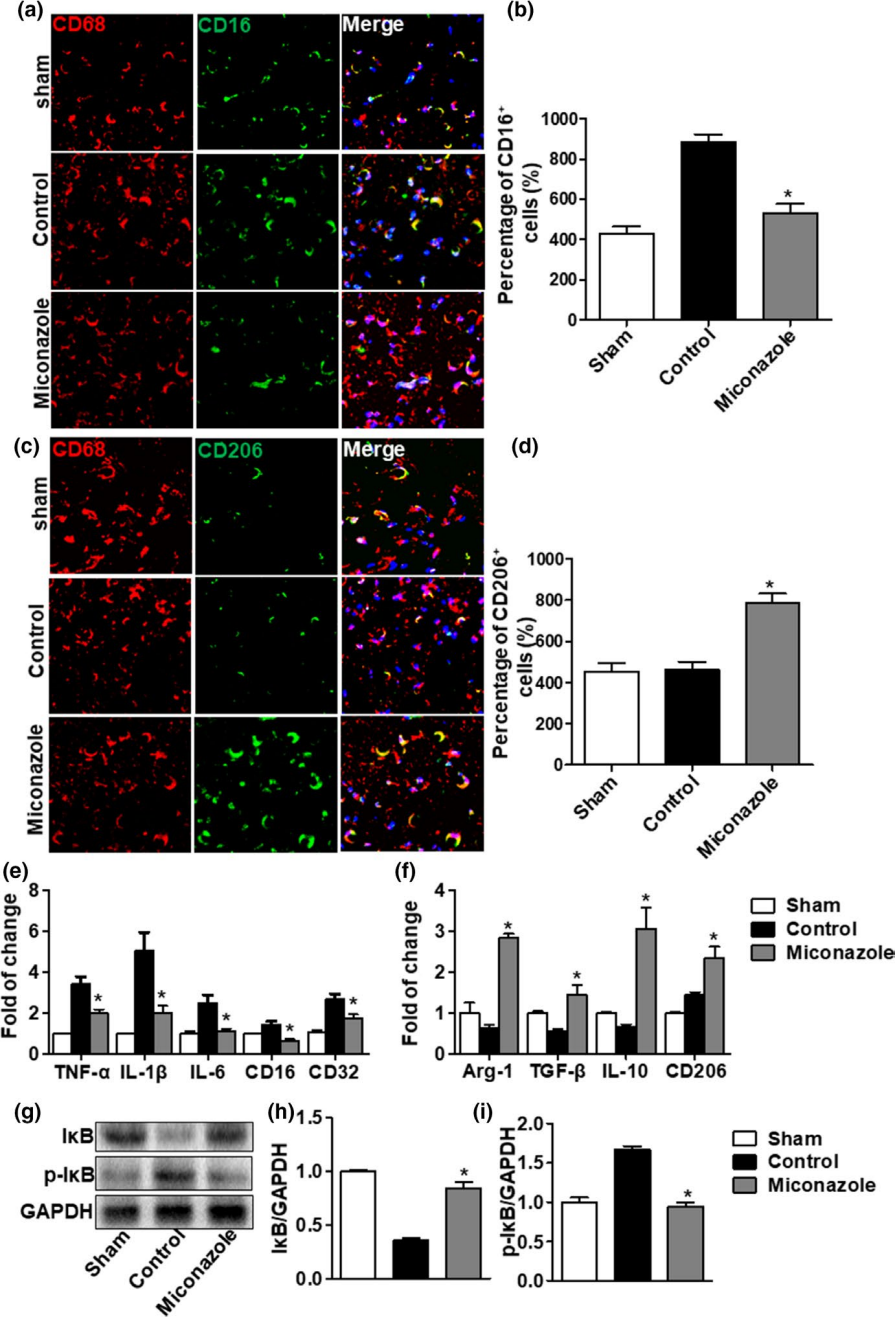
Miconazole promotes M2 macrophage polarization in vivo. (a, c) Fluorescence photomicrographs showing M1 and M2 macrophages in the sciatic nerves of injured mice. Tissue sections were immunostained for markers of M1 and M2 macrophages as indicated. Scale bar indicates 10 μm. (b) Quantitation of immunohistochemistry. Counts per view of CD16+/Iba + cells (M1 phenotype) in the sciatic nerve showed that miconazole reduced the number of M1 macrophages. (d) Counts per view of CD206+/Iba + cells (M2 phenotype) showed that miconazole increased the number of M2 macrophages. Comparisons to control group were performed using the Mann–Whitney *U* test; quantitation shows the means ± *SEM* (*p < .05; *n* = 6). The experiment was repeated three times with similar results. (e) qRT‐PCR for mRNA expression of M1 cytokines (TNF‐α, IL‐1β, IL‐6, CD16 and CD32) in crushed nerves**.** The gene expression of proinflammatory cytokines was decreased in treatment groups. (*n* = 6 per group). (f) qRT‐PCR for mRNA expression of M2 cytokines (Arg‐1, CD206, TGF‐β IL‐10) in the injured nerve showed increases in treatment groups (*n* = 6 per group). (g–i) Expression of IκB and p‐IκB was detected using immunoblotting. The data are expressed as the means ± *SEM*. *p < .05 versus control group

To investigate the potential mechanism of the favorable effects of miconazole, we investigated the possible changes in macrophage of sciatic nerves. In sciatic nerves harvested on day 7 postinjury and subjected to double immunofluorescence staining, the expression of CD16/32 was strongly present in Iba1^+^ cells in the control group, while CD16/32 expression was significantly decreased in the miconazole treatment group (*p* < .05; Figure 3a,b). In addition, the expression of the M2 markers CD206 was higher in the miconazole treatment group compared with the control group (*p* < .05; Figure 3c,d).

After nerve crush injury, series of inflammatory cytokines released from immune cells and accumulated to the injury site. Here, we tested the mRNA expression of multiple inflammatory cytokines released by M1 macrophages (IL‐1β, TNF‐α, IL‐6, and CD16, CD32) in the sciatic nerve at day 7 after injury (Figure 3e,f). Overall, the levels of cytokine expression increased following PNI compared to the sham group. However, in the miconazole group, these increases in expression were reduced compared to the increases observed in the control group (Figure 3e,f). TNF‐α, IL‐6, and IL‐1β are proinflammatory cytokines that may aggravate nerve injury. In contrast, TGF‐β and IL‐10 are anti‐inflammatory cytokines, as they can reduce inflammatory reactions (Ydens et al., 2012). In the control group, IL‐10 expression increased by 2.6‐fold at 7 days after injury compared to the sham‐operated group (Figure 3f). Miconazole treatment further increased IL‐10 expression compared to that of the control group (Figure 3f). We also found that a higher level of TGF‐β expression in the miconazole treatment group at 7 days compared with the control group (Figure 3f). In addition, the M2 state mRNA markers (CD206 and Arg 1) were induced dramatically at day 7 postinjury (Figure 3f). These data suggest that miconazole plays a role in the phenotypic transformation of macrophages, with the result that primary macrophages are converted to anti‐inflammatory phenotypes.

### 3.4 Effects of miconazole on activation of the NF‐κB pathway in PNI mice

Alterations in the levels of cytoplasmic p‐IκB/IκB proteins in the sciatic nerve after crush are shown in Figure 3. The activity of IκB was estimated by the ratio of p‐IκB to IκB, as the phosphorylation of IκB is important for NFκB activation. We observed a marked increase in the ratio of p‐IκB/IκB in the sciatic nerve after injury contrasted with the sham group. Miconazole markedly reduced IκB phosphorylation at day 7 after injury (Figure 3g–i). Thus, miconazole inhibits the phosphorylation of IκB, and this results in the inhibition of downstream components. In our hands, miconazole treatment suppressed the activity of IκB as well as decreased the M1 macrophage phenotype. (IκB: 0.68 ± 0.32 in the miconazole treatment group vs. 0.42 ± 0.20 in the control group, *n* = 6 per group; p‐IκB: 0.59 ± 0.24 in the miconazole treatment group vs. 1.65 ± 0.14 in the control group, *p* < .05, *n* = 6 per group). This implies that miconazole may shift the polarization of macrophages by inhibiting IκB activation.

### 3.5 Effects of miconazole on macrophage polarization in vitro

**Figure 4:**
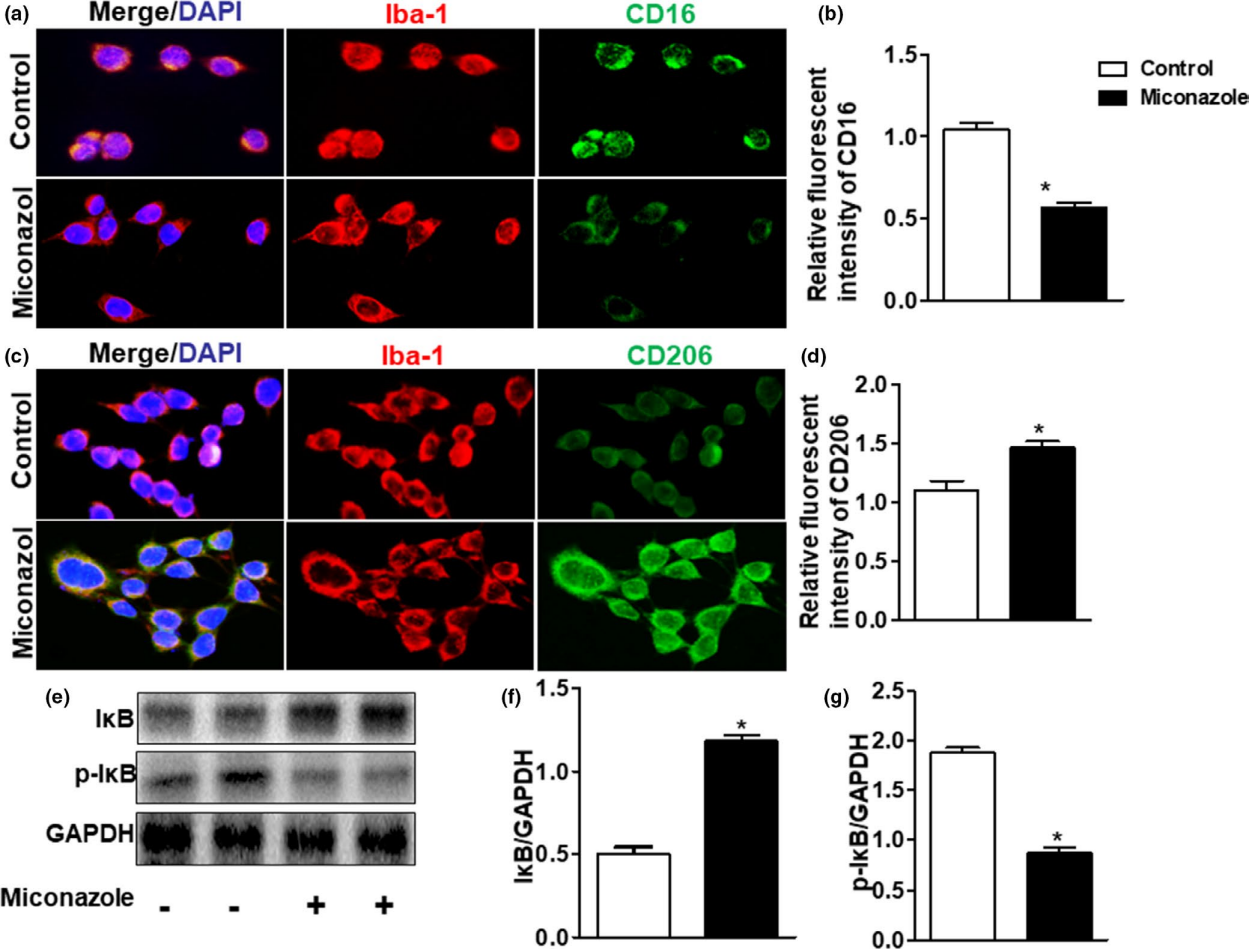
Miconazole suppresses the polarization of M1 macrophages and promotes the M2 phenotype in vitro. Representative images of the proportion of M1 (a) or M2 (c) phenotype cells in each group. RAW246.7 cells were cultured in growth medium with LPS (100 ng/ml) or IL‐4 (20 ng/ml). The phenotype of RAW246.7 cells was examined by the co‐ expression of the M1 marker CD16/32 (green) and the M2 marker CD206 (green) with the macrophage marker Iba‐1 (red). M1 polarization was inhibited by miconazole, but miconazole also enhanced M2 polarization (*n* = 3 per group). (b) and (d) Statistics for the RAW246.7 phenotypes in the presence or absence of miconazole. (e–g) Expression of IκB and p‐IκB was detected using immunoblotting (*n* = 3 per group). The data are expressed as the means ± *SEM*. *p < .05 versus control group

To further confirm the effect of miconazole on the polarization of macrophages, a polarization experiment was carried out in RAW246.7 cells with or without miconazole. RAW246.7 cells were cultivated in medium containing LPS (100 ng/ml) or IL‐4 polarization‐inducing cytokines in the presence of miconazole. After 12 hr of polarization, a significant change in the amount of M2 macrophages was observed in the miconazole‐treated group compared with the control group, while M1 polarization was suppressed by miconazole (Figure 4a–d). These results offered further evidence to prove that miconazole influences macrophage polarization.

The transcriptional activity of IκB during polarization was also examined. Our data showed that IκB was decreased and p‐IκB was increased along with the increased M1 phenotype trigged by LPS treatment. After intervention with miconazole, IκB was increased and p‐IκB was decreased dramatically (Figure 4e–g). (IκB: 0.54 ± 0.32 in the control group vs. 1.23 ± 0.08 in the miconazole treatment group, *p* < .05, *n* = 3 per group; p‐IκB: 1.83 ± 0.16 in the control group vs. 0.75 ± 0.31 in the miconazole treatment group, *p* < .05, *n* = 3 per group). These results demonstrate that miconazole regulates the polarization of macrophages through IKB activation.

## 4 DISCUSSION

It is a recognized viewpoint that peripheral neurons could regenerate but central neurons do not (Zochodne, 2012). The neuropathy caused by injury extends far beyond traumatic lesions. According to related reports, approximately 2.8% of peripheral nerve injuries occur in patients with multiple trauma, moreover 5% of root and plexus lesions (Zochodne, 2012). Peripheral nerve damage is often hard to recover, and clinical injuries are often more complex, so it is essential to investigate new therapies for peripheral nerve injury. In recent decades, local inflammation has been realized playing a vital role in peripheral nerve injury (Bombeiro et al., 2016; Chen, Piao, et al., 2015; Scheib & Hoke, 2016). Peripheral nerve injury is a complex pathologic process involving multiple steps, perhaps the most important of which is Wallerian degeneration. Wallerian degeneration induces multiple changes including glial cell proliferation, axonal degeneration, myelin disintegration, blood–nerve barrier (BNB) compromise, even the infiltration and activation of immune cells (Camara‐Lemarroy, Guzman‐de la Garza, & Fernandez‐Garza, 2010). In recent decades, macrophages have been important for peripheral nerve injury (Bombeiro et al., 2016; Chen, Piao, et al., 2015; Scheib & Hoke, 2016). Macrophages are resident immune cells and account for 2%–9% in peripheral nerves and are in the business of phagocytic abilities (DeFrancesco‐Lisowitz et al., 2015). Previous studies have shown that in animal models of sciatic nerve injury, macrophages infiltrated into the injured nerves at the beginning of 2 or 3 days after injury and peaks at 7 days postinjury (Mueller et al., 2001). These resident macrophages could phagocytose myelin debris in the early phase of injury and have been traditionally considered to have harmful effects, but recent studies are beginning to demonstrate that they play a crucial role in peripheral nerve regeneration (Chen, Cescon, et al., 2015). Since macrophages are highly plastic, the local microenvironment may have impact on the phenotype of macrophage. The macrophage activation spectrum consists of proinflammatory M1 macrophages which are referred to as “classically activated,” and prorepair M2 macrophages referred to as “alternatively activated” (Martinez & Gordon, 2014). Macrophages could be divided into two main subtypes which refer as classically activated macrophage (M1 type) and alternatively activated macrophage (M2 type; Kwon et al., 2017). The classical activated macrophage could produce proinflammatory cytokines and exacerbate tissue damage while the alternatively activated macrophage could promote tissue repair through anti‐inflammatory cytokines (Saha, Shalova, & Biswas, 2017). M2 macrophages downregulate inflammatory mediators, but upregulate the expression of anti‐inflammatory factors. Akt, mTOR, NF‐κB and STAT6 pathway activation are involved in the macrophage polarization (Czimmerer et al., 2018; Lin et al., 2018; Xiong, Liu, & Yang, 2016).

The polarization states of macrophages play different roles in repair and remyelination in the peripheral nervous system (Brown, Ratner, Goodman, Amar, & Badylak, 2012; Chen & Bonaldo, 2013; Gordon & Taylor, 2005). High levels of M1‐polarized cells suggest more sever of peak demyelination, and M2‐polarized macrophages may improve remyelination, neuronal preservation and functional outcomes (Ma et al., 2015; Mikita et al., 2011). Therapies that shift the balance in favor of the M2 polarization axis may benefit clinical outcomes in peripheral nerve injury.

Miconazole is an antifungal medication related to fluconazole (Diflucan), which is used dermatologically or vaginally to treat fungal infections (Ahmed, El‐Say, Mahmoud, Samy, & Badawi, 2012; Buechner, 2014). Miconazole prevents important substances required for the growth and function of fungal organisms. Miconazole was authorized by the FDA in 1974. In recent years, studies have demonstrated that miconazole is effective in promoting precocious myelination in the central nervous system of a mouse model of experimental autoimmune encephalomyelitis (EAE; Najm et al., 2015). In our study, we confirm that miconazole has a protective role in sciatic nerve crush injury by shifting macrophage differentiation from the detrimental state to the restorative state, an effect fully in accord with our aim of identifying compounds that promote recovery after sciatic nerve injury.

Our study revealed that miconazole treatment diminishes the secretion of proinflammatory cytokines but enhances that of anti‐inflammatory cytokines at day 7 postinjury. Proinflammatory cytokines such as IL‐6, IL‐1β, and TNF‐α contribute to the expansion of the sciatic nerve crush (Day et al., 2014; Liou et al., 2013; Pajer et al., 2014; Sacerdote et al., 2013). In contrast, IL‐10 and TGF‐β are pleiotropic immunoregulatory cytokines that play a key role in the anti‐inflammatory effect associated with tissue recovery (Sacerdote et al., 2013; Ydens et al., 2012). We found that miconazole treatment can abate the degree of proinflammation production but increase the levels of TGF‐β and IL‐10 mRNA at day 7 postsciatic nerve injury. Our results demonstrate that treatment with miconazole improves tissue repair by promoting the resolution of inflammation and by influencing direct cytoprotective effects in the sciatic nerve.

**Figure 5:**
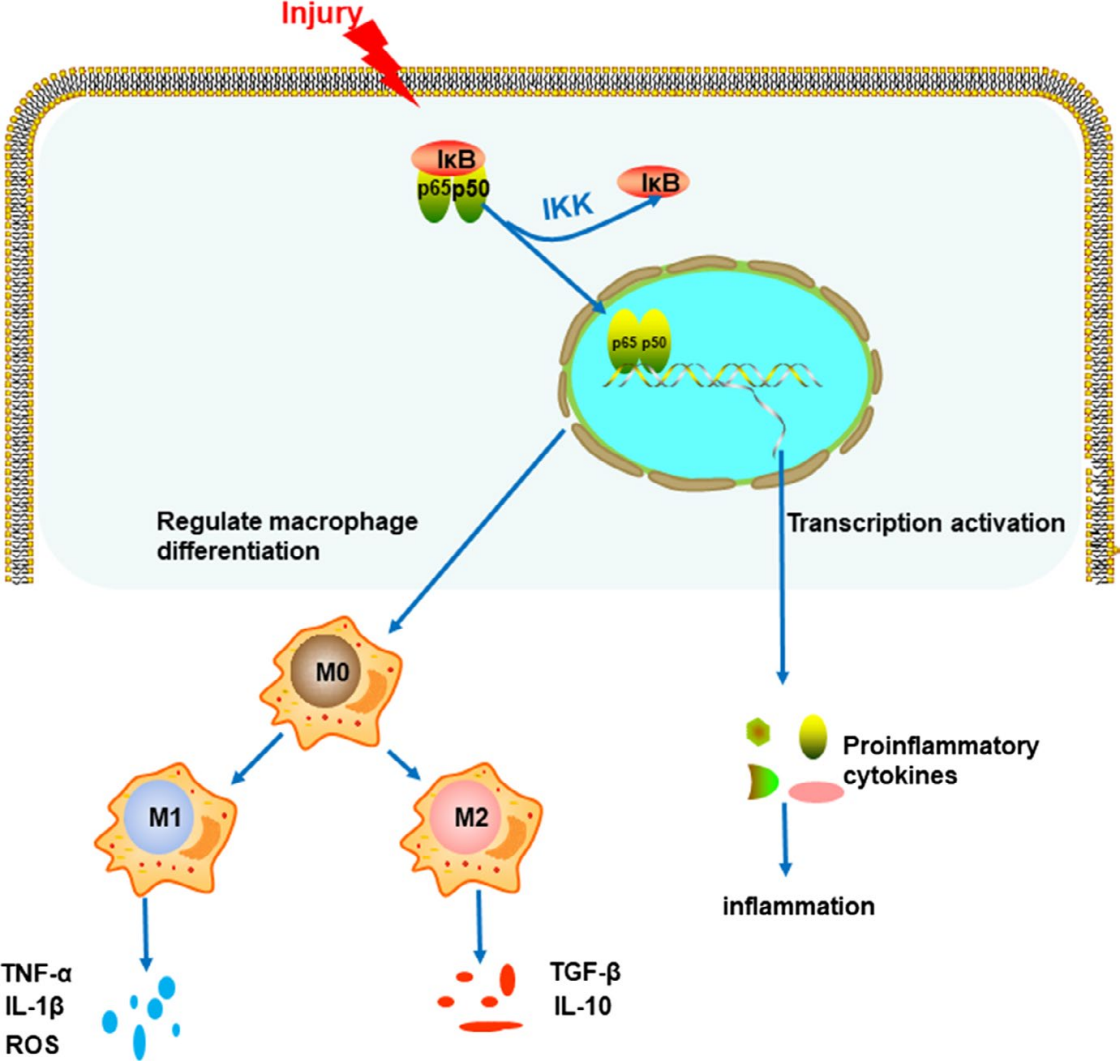
Schematic diagram of the effect of miconazole on nerve damage postcrush. Miconazole exerts neuroprotection by phenotypic modulation of the macrophage shift and suppression of NF‐κB signaling. Miconazole inhibits IκB phosphorylation and decreases NF‐κB translocation into nuclei, which activates downstream gene transcription. Thus, expression of proinflammatory cytokines is suppressed and the polarization of macrophages shifts to the M2 phenotype

A large number of studies have confirmed that NF‐κB pathway plays a proinflammatory mechanism in promoting the expression of macrophages and can regulate the differentiation of macrophages into M1 type (Chen et al., 2017; Krausgruber et al., 2011). Finally, our results also show that miconazole treatment decreases the p‐IκB/IκB ratio in crushed sciatic nerves, suggesting a link between macrophage polarization and suppression of IκB activation. Thus, miconazole can be demonstrated to regulate the differentiation phenotype of macrophages through the NF‐κB pathway, transforming macrophages to the anti‐inflammatory M2 phenotype. Additional, M2 macrophages play an active role in promoting PNI regeneration. To confirm this hypothesis, we conducted in vitro experiments, which agreed with the in vivo results. A previous study also supports this result (Jiang, Westerterp, Wang, Zhu, & Ai, 2014). However, further investigation is required to fully uncover the underlying mechanisms through which miconazole acts on peripheral nerve injury. There are also some limitations of our study. Conditional transgenic or knockdown mice were not used in the study and lineage‐tracing study would boast our finding. Also, we only analyzed one time point after PNI and the longer follow‐up period analysis would boast our finding. Based on the above experimental conclusions, Figure 5 was designed and produced, which vividly demonstrates the different polarization directions of macrophages mediated by the NF‐κB pathway.

In conclusion, our study demonstrates that miconazole induces a shift in macrophage phenotype, inhibits inflammatory responses, reduces myelinoclasis, and improves neurological function in a sciatic nerve injury model, and these effects may be mediated by the NF‐κB pathway. Overall, our findings may have implications for developing a therapeutic strategy that benefits human peripheral injury.

## CONFLICT OF INTEREST

The authors declare no conflict of interest.

## AUTHOR CONTRIBUTIONS

Liangliang Zhang, Zengyun Liu and Xiuju Chen designed the experiments for the project and wrote the paper. Zengyun Liu, Zhen Tian, Liguo Tang, acquired and analyzed the data. Qingluan Han, Hui Xu, Cunmin Rong and Zhiyong Ren revised the paper. All authors reviewed and approved the final manuscript.

## Data Availability

The data used to support the findings of this study are available from the corresponding author upon request.
